# Hidden pleiotropy of agronomic traits uncovered by *CRISPR-Cas9* mutagenesis of the tyrosinase CuA-binding domain of the *polyphenol oxidase 2* of eggplant

**DOI:** 10.1007/s00299-023-02987-x

**Published:** 2023-02-02

**Authors:** Preshobha Kodackattumannil, Geetha Lekshmi, Martin Kottackal, Shina Sasi, Saranya Krishnan, Salima Al Senaani, Khaled M. A. Amiri

**Affiliations:** Khalifa Center for Genetic Engineering and Biotechnology, United Arab Emirates University, P.O. Box. 15551, Al Ain, United Arab Emirates

Crop improvement relies on agronomic trait variations. Pleiotropy defines as a spectrum of phenotypic traits controlled by a single gene, i.e., divergent functions of a single gene at the phenotypic level. Divergence in gene function is caused by mutations in coding regions, resulting in amino acid changes that modify protein function, that interact in complex ways to determine domains and levels of expression in different tissues throughout development (Hendelman et al. 2021). Divergent gene function shaped by mutation, gene duplication, and gene loss is a hallmark of evolution (Holland et al. 2017). Crop-up of several phenotypes by mutation indicates hidden pleiotropy as a feature of past evolution and is significant in crop improvement.

*Solanum melongena* L. (Eggplant), an Old-World crop has been listed by the FAO as the fourth largest vegetable crop. Browning of freshly cut fruits negatively affects its commercial value. Polyphenol oxidase is the key player in the enzymatic browning of fruits and vegetables (Hamdan et al. 2022). *PPO*, a nuclear gene for chloroplast products is compartmentalized in plastids. During tissue disruption, PPO interacts with phenolic substrates localized in the vacuoles and results in browning. The *PPO* genes of eggplant are in clusters on chromosome 8 (Fig. 1a), except for *PPO10,* which is mapped to chromosome 2 (Maioli et al. 2020). *PPO2* belonging to the tyrosinase group binds two copper ions (CuA and CuB), and the six conserved histidine residues coordinate the two copper-binding sites (Solomon et al. 2001). *PPO2* (MN999539.1) is an intronless-gene encoding a 594 aa (1785 bp) protein with a thylakoid luminal transfer peptide containing two copper-binding domains on the negative strand (Maioli et al. 2020). CRISPR/Cas9-mutagenesis is a tool to improve agronomic traits-of-interest (Beracochea et al. 2022). Here, we demonstrate the potential of CRISPR/Cas9 mutagenesis in pleiotropy, by editing the CuA-domain of the *PPO2* gene for the first time.

**Fig. 1:**
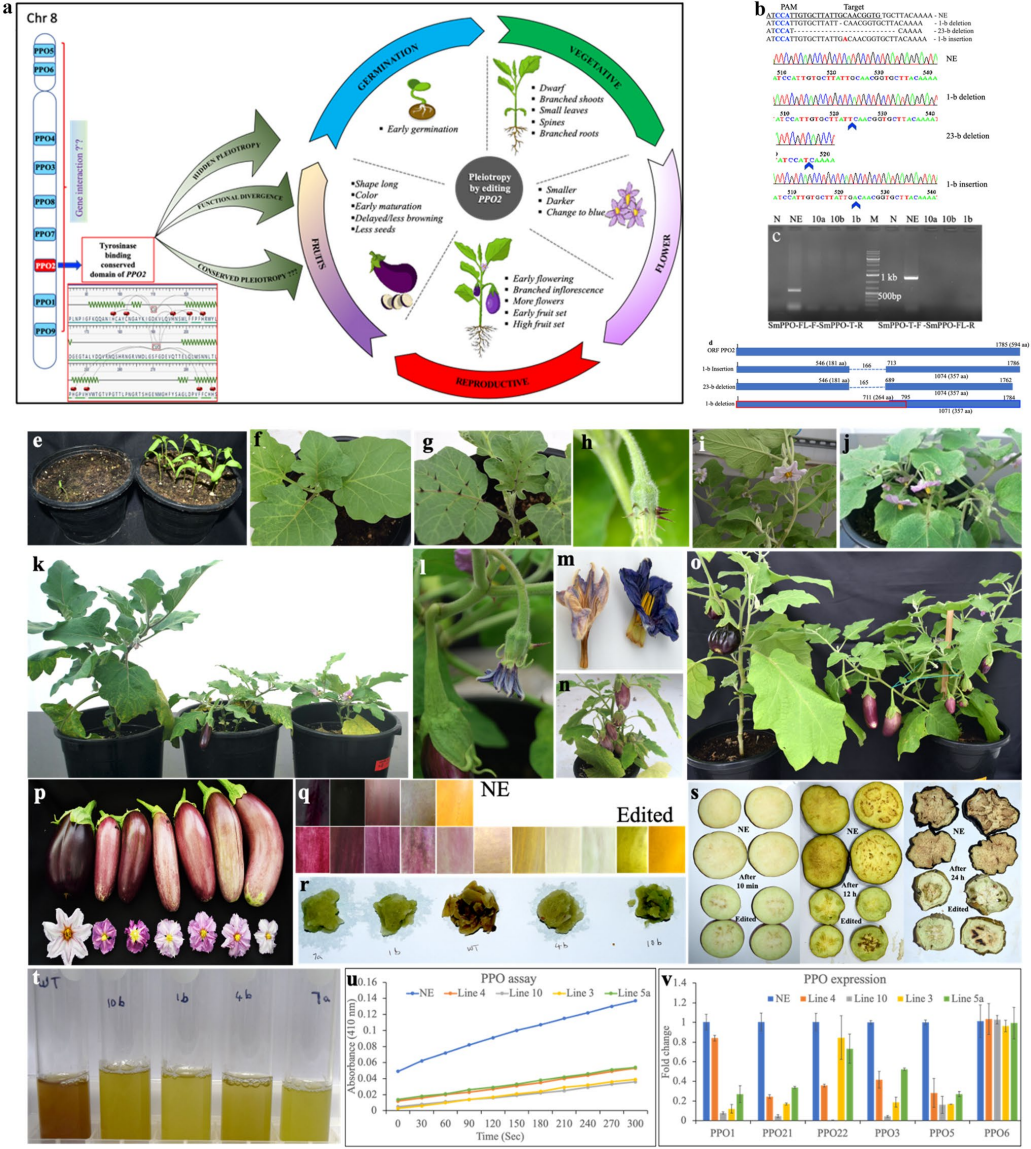
**a** Graphical representation of pleiotropy in eggplant revealed by editing the *PPO2*; **b** types of editing of the target; **c** confirmation of the 23-b-edited lines. **d** ORFs of *PPO2* by different types of editing; **e-t** phenotypes of *PPO2-*edited eggplant. **e** Germination (Left NE, Right Edited); **f** leaves of NE with no spines; **g** spines on edited-plant; **h** spines on calyx; **i** solitary flowers on NE; **j** branched inflorescence on edited plant; **k** NE (left) and edited plant showing dwarf and early flowering/fruiting; **l** senescening flower (blue); **m** senescent flower of NE (left) and edited (right) plant; **n** edited plant with fruits on branched inflorescence; **o** edited plant (right) with branches and subbranches and more fruits than NE (plants at the same age); **p** mature fruits and flowers of NE (Left) and edited-lines; **q** fruit color panel of NE and edited plants from fruit-set to ripening; **r** nonbrowning of the scraped flesh of edited (10b, 1b-23-base-deleted; 4b-1-base-inserted, 7a-1-base-deleted) and browning of NE (middle); **s** browning of NE and nonbrowning of edited fruit-slices up to 24 h; **t** browning of fruit juice of NE (left) and edited plants showing no browning (5 min); **u** PPO assay of the different edited-lines; **v** relative expression of the *PPO2* and other *PPO* genes. The relative expressions except *PPO6* are significantly different (*P* < 0.05) in NE and edited-lines. *PPO21* (prior to CuA-binding) and *PPO22* (after the CuA-binding) are different primers of *PPO2* (NE- Non-Edited; Line 3 and 5a—1-b-deleted; Line 4—1-b-inserted; Line 10—23-b-deleted; M-1 kb-plus marker; *N*-negative control; a, b are biological replicates) (color figure online)

The frequency of genome-editing was 64.7% (11 out of 17 regenerated). Mutagenesis of the CuA-binding domain of the *PPO2* occurred on the negative strand was three types: 1-base insertion, 1-base, and 23-base deletion (Fig. 1b; Table S1). Of the GE_3_ plants, lines 5 and 7 (Table S1) were Cas9-free (Fig. S1a), while lines 5a, and 9 (Table S1) were marker-free (Fig. S1b). The line 5a was Cas9- and marker-free (Fg. S1a, b).

Although no off-target was observed by Cas-OFFinder (http://www.rgenome.net/cas-offinder/), we confirmed the 23-base knockouts using PCR (Fig. 1c). As an intronless gene, the amplification of the DNA with forward primer from the start of the full-ORF with deleted 23-b as a reverse primer and deleted 23-b as a forward primer with the primer from the C-terminal end of full-ORF as the reverse primer (Table S2) produced no amplicon against 548 and 1254 bp in the NE (Fig. 1c).

The mutagenesis at the CuA-binding domain resulted in two ORFs (Fig. 1d). In all cases, the CuA- domain, which binds two copper ions via three histidines (H178, H196, H205), was abolished (Fig. S2c–m). The amino acid sequence showed fewer histidine residues, which are significant for binding two copper ions (Fig. S1c, h–l). The ORF after the edited region was homologous in all types of editing (Fig. S1n). However, the mutation of the CuA-binding domain and truncation of the ORF changed the binding specificity of the CuB-domain (Fig. S1l). The removal of the histidines (H326, H330, H368) affected the coordination of the CuA- and CuB-binding domains (Fig. S1c–m).

Mutagenesis of the *PPO2* gene uncovered a spectrum of phenotypes: early seed germination, small leaf size, no leaf pubescence, plant dwarfism, more branching, highly branched roots, branched inflorescences, more flowers, high fruit set, bluish corolla of the senescing flower, thorns on the stem, prominent spines (on the petiole, lamina, and calyx), differences in fruit color, length, shape, and weight (very prominent features), reduced fruit maturation time, reduced and delayed browning of fruit-cuts (also in fruit-scoops and juice), and fewer seeds (Fig. 1a, e–u; Table S3). Traits such as plant dwarfing, color, shape, and weight, early flowering, a high number of flowers and early fruit set and maturation, fewer seeds, and reduced and delayed browning of fruits are agronomically important. Unlike NE plants, the edited plants continued to produce more flowers and fruits even after 120 days. The fruit weight of the 1-base-insertion lines was lower but produced more fruits (Table S3). The spines and thorns were abundant on 23-base-edited lines (Table S3).

The color of the corolla and fruits of the mutants exhibited differences from that of NE (Fig. 1p). The fruit color of the edited lines, initially purple compared to the dark purple (almost black) of the NE, changed to light purple (Fig. 1p). The fruit color from fruit set to ripening exhibited differences in NE and edited lines (Fig. 1q), which signifies the role of *PPO2* in fruit ripening.

Fresh-cut fruit slices of the 1-base-insertion and 1- and 23-base-knockout lines incubated at 22 ± 1 °C and 37 ± 1 °C showed no browning in two hours, and they looked almost the same after 24 h (Fig. 1s, Fig. S2a, b). Browning was visible within 10 min in NE fruit slices (Fig. 1s; Fig. S2a, b). The browning of the fruit scoops and juice extract of the NE plants was visible in 5 min and became dark brown in 10 min, while those of the mutants remained greenish-yellow even after an hour (Fig. 1r, t). The browning assay revealed high activity in NE fruits and the weakest browning on 23-base knockouts (Fig. 1u). The fruits of mutants showed 77%, 66%, and 79% less PPO activity in 1-b insertion, 1-b deletion, and 23-b deletion, respectively, over NE in five min. Simultaneous editing of eggplant *PPO* genes, viz., *PPO3*, *PPO4*, and *PPO5* by Maioli et al. (2020) reported 48–61% reduced browning compared to control, but with no phenotypic variations even in fruit characters. The CRISPR mutagenesis of *StPPO2* gene in potato displayed 73% reduced enzymatic browning (González et al. 2020).

Relative expression of *PPO2* genes confirmed reduced browning in the edited plants, and was lowest in 23-base-edited (Fig. 1v). The expression of the different *PPOs* in mutants was lower than those in NE plants, except in the case of *PPO6,* which showed no significant difference (Fig. 1v).

A phylogenetic analysis of the CuA-binding domain of *PPO2* in different species of Solanaceae showed it to be conserved (Fig. S3a, b). As the eggplant is assumed to be derived from the wild African species *S. incanum,* the resurgence of spines on the *PPO2*-edited plants as in *S. incanum,* indicates the loss of function during domestication.

In summary, the results reveal hidden pleiotropy of the PPO gene. Pleiotropy by genome editing becomes a useful tool to speed up breeding programs.

## Supplementary Information

Below is the link to the electronic supplementary material.

Supplementary file1 (DOCX 24 KB)

Supplementary file2 (PPTX 3014 KB)

## Data Availability

The data is available on request to correspondent author.
