# Supplementary material for: Hidden pleiotropy of agronomic traits uncovered by CRISPR-Cas9 mutagenesis of the tyrosinase CuA-binding domain of the polyphenol oxidase 2 of eggplant
Source: Plant Cell Rep. 2023 Feb 2;42(4):825–8. doi: 10.1007/s00299-023-02987-x (PMC10119049; doi:10.1007/s00299-023-02987-x)
Supplement: Supplementary file 1 — Supplementary file1 (DOCX 24 KB) [file 299_2023_2987_MOESM1_ESM.docx]

***Suppl. Methods 1***

**Hidden pleiotropy of agronomic traits uncovered by *CRISPR-Cas9* mutagenesis of the tyrosinase CuA-binding domain of the *polyphenol oxidase 2* of eggplant**

Preshobha Kodackattumannil^*^, Geetha Lekshmi^*^, Martin Kottackal^**^, Shina Sasi, Saranya Krishnan SR, Salima Saeed Rashed Humaid Alsenaani, Khaled MA Amiri^**^

**Plant material**

*Solanum melongena* L. *var*. Black Beauty was used for genome editing experiments. Black beauty is a top-notch popular heirloom nonhybrid variety of eggplant with a teardrop-shaped (oval), glossy, purplish-black fruit.

**Plasmid construction**

A twenty-base target sequence for editing the *PPO2* gene was designed from the sequence retrieved from MN999539.1 (NCBI) using CRISPRdirect (<https://crispr.dbcls.jp>). The 20-base target of the CuA-binding domain (from base 532 to 615) of the gene was synthesized with adaptors on the forward and reverse primers (Table S2) and inserted into the Cas9 (under the 35S promoter) vector pHSE401 (<https://www.addgene.org/62201/>) carrying kanamycin for bacterial selection and hygromycin for plant selection. The prepared target duplex was inserted into the vector using the Goldengate protocol (NEB) following the manufacturer’s instructions. Target-inserted colonies after transformation to DH5α were selected on LB plates with kanamycin by colony PCR using the M13 forward and reverse primers of the target, yielding an amplicon of 400 bp. The target insert was confirmed by sequencing the plasmid extracted from the positive colonies using a plasmid extraction kit (Qiagen). The confirmed plasmid was introduced into the *Agrobacterium tumefaciens* strain EHA 105 using the electroporation method. The positive colonies were confirmed and used to transform the cotyledon explants.

**Plant transformation, selection, and regeneration**

Cotyledon explants of (1-1.5 cm) excised from 15-day-old seedlings of eggplant (*Solanum melongena*) *var*. Black beauty germinated on half-strength MS (Murashige and Skoog, 1962) medium (2% sucrose) infected with *Agrobacterium tumefaciens* EHA105 harboring the pHSE401 vector with the editing target for the *PPO2* gene (Table S2) in MS liquid medium (3% sucrose) containing 200 μM acetosyringone after incubation for 20 min on a shaker (50 rpm) at RT followed by 20 s of sonication and 10 min of vacuum infiltration. Blot-dried agroinfected explants were incubated on acetosyringone (200 μM) added to MS1 medium (MS + 4.6 μM trans-zeatin and 2.3 μM thidiazuron) in the dark. After being washed with timentin (300 mg l^-1^) solution, the infected explants were cultured on MS1 medium supplemented with Timentin (300 mg l^-1^) and hygromycin (25 mg l^-1^) and incubated in 16 h light:8 h dark, with 40-50 μmol m-2 s-1 intensity and a temperature of 25±2 °C. The developed shoots were transferred onto MS medium (1.5% maltose) with 8.88 μM benzyladenine and 0.58 μM gibberellic acid (GA_3_) and subsequently onto MS medium (1% maltose) with 1.1 μM kinetin, 0.3 μM GA_3_ and 3 mg l^-1^ silver nitrate. The well-grown shoots were rooted in a half-strength MS medium containing 0.5 μM α-naphthaleneacetic acid. All media were added with hygromycin and Timentin. GE_3_ plants of the edited lines were selected using hygromycin. The edited and nonedited (NE) plants were grown following a randomized design with 5 replicates and compared. PCR using the DNA extracted from edited and NE plants was used to amplify the *PPO2* gene (Table S2), and the editing was confirmed by sequencing three replicates of each line. The MS medium used was from Phytotech Laboratories, USA, and the chemicals were from Sigma‒Aldrich, USA.

**Screening of genome-edited lines**

Genomic DNA was extracted from the leaves of rooted shoots growing on half-strength MS medium (with 2% sucrose) containing 100 mg l-1 timentin and 25 mg l-1 hygromycin using the cetyltrimethylammonium bromide method (Dutta *et al.,* 2013). The target regions were amplified with sequence-specific primers for direct Sanger sequencing (Table S2). PCRs (50 μl) were performed with Phusion Hi-Fidelity polymerase of NEB using the forward and reverse primers and 100 ng of DNA template following the manufacturer's protocol. The PCR conditions consisted of 98 °C for 30 s, 30 cycles of 98 °C for 10 s, 60 °C for 20 s, and 72 °C for 30 s and a final extension of 72 °C for 5 min. The DNA isolated from NE plants was used as the positive control. Amplicons were extracted from the gel using a gel extraction kit (Qiagen). The above procedure was carried out with GE_3_ plants to confirm homozygous editing.

**Acclimatization of GE_0_ plants**

PCR-confirmed well-rooted shoots, after being cleared of agar particles, were planted in small pots containing a soil mix (sand and peat; 1:1). The pots were initially covered by a polyethylene bag for 10 days to retain moisture during acclimatization in the plant room (25±2 °C; 70% humidity; 400 μmol/m^2^/s - Heliospectra LED Lights). The plantlets were subsequently transplanted into 5 L pots and grown in a greenhouse until seed harvest (30±2 °C; 70% humidity; 400 μmol/m^2^/s – Heliospectra LED Lights). The nonedited and edited plants were fertilized by adding 2 g/l NPK (20:20:20) biweekly until the onset of flowering and thereafter NPK (12:14:24) at biweekly intervals.

**Off-target analysis and confirmation of editing**

Off-target analysis was carried out using Cas-OFFinder (<http://www.rgenome.net/cas-offinder/>) and specific analysis of Chr 8 and other *PPO* genes (which were also checked manually). The editing of the 23-base knockouts (GE_3_) was confirmed using PCR. The PCR was carried out using the DNA of 23-base knockouts and the forward primer of the start codon of the full ORF (SmPPO-FL-F) with 23 bases deleted as the reverse primer (SmPPO-T-R) and 23 bases deleted as the forward primer (SmPPO-T-F) with the reverse primer (SmPPO-FL-R) of the coding (stop codon of full ORF) region (Table S2).

**Generation of GE_2_ and GE_3_ plants**

The edited GE_1_ seeds collected from different GE_0_ plant lines were surface sterilized separately by treatment with 10% Clorox solution in 2 ml Eppendorf tubes for 10 min with inversion. After being washed three times with sterile water, the blot-dried seeds were cultured on half-strength MS (2% sucrose) containing 25 mg l-1 hygromycin for germination. The healthy plants derived from GE_1_ seeds after 20 days were transplanted into the soil as described previously. GE_2_ seeds germinated on hygromycin-containing half-strength MS were subsequently transplanted into soil, and the seeds were collected from mature fruits to raise the GE_3_ plants.

**Phenotyping of edited plants**

All phenotypic features from seed germination to seed set, *i.e.,* vegetative to reproductive traits, were documented for GE_0_ to GE_3_ plants. Only the stable traits that appeared in the GE_3_ generation were considered phenotypes. The measurement-requiring features were documented from 5 replicates.

**Bioinformatic analyses**

ORF analysis was accomplished using ORF-Finder (NCBI). Multiple sequence analyses of the truncated proteins were performed by Clustal Omega (<https://www.ebi.ac.uk/Tools/msa/clustalo/>). Phylogenic trees were constructed using the aligned sequences. The amino acid composition, isoelectric point, molecular mass, and absorption coefficient of the truncated proteins were analyzed by Prot Pi (<https://www.protpi.ch>). A conserved domain search was performed using the Conserved domain search of NCBI <https://www.ncbi.nlm.nih.gov/Structure/cdd/wrpsb.cgi>), and binding sites were predicted using DNASTAR (<https://www.dnastar.com>).

**Browning assay**

Fresh-cut fruit circles (0.5-cm-thick circles) of the edited (GE_3_ plant fruits) and nonedited (NE) lines at the harvest stage and mature stage were incubated at room temperature (22±1 °C and at 37 °C) for up to 24 h for visual observation of browning. The browning of fruit juices from edited and NE lines incubated at 22±1 °C was visually observed until 24 h.

**Enzyme extraction and PPO activity determination**

PPO was extracted by homogenizing fruits of NE and edited (GE_3_) plants at the vegetable harvest stage, and activity was determined as reported previously (Arnnok *et al.,* 2010). One gram of powdered sample was homogenized with 1 ml of 100 mM potassium phosphate extraction buffer (pH 7.0) containing 2 mM EDTA ferric sodium salt and 2% (w/v) polyvinylpyrrolidone. Following centrifugation of the homogenate in a precooled microcentrifuge at 9000 rpm for 30 min, the supernatant was aliquoted into 1.5 mL microcentrifuge tubes and stored at -20 °C for later use in assays. For the PPO assay, the contents of the assay mixture (3 mL), namely, phosphate buffer solution (pH 7.0, 100 mM, 1.95 ml), 1 mL of 100 mM catechol as a substrate, and 50 μl of the enzyme extract were mixed in a test tube. The mixture was transferred immediately to a 10-mm path-length cuvette. The absorbance at 410 nm was recorded continuously at 25 °C every 30 s for 5 min using a spectrophotometer (Thermo Scientific).

**RNA extraction and expression analysis**

The fruit flesh (vegetable harvest stage including the skin) of the edited (GE_3_ plants) and NE plants after 15 min of slicing was ground in liquid nitrogen to a fine powder. Total RNA was extracted using the Maxwell® RSC Plant RNA Kit (Promega, Madison, USA). The quantity and quality of RNA were checked by Nanodrop 2000 (Thermo Scientific) and gel electrophoresis, respectively. cDNA was synthesized from 1 μg total RNA using a QuantiTect Reverse Transcription kit (Qiagen, Germany) following the manufacturer’s instructions. qRT-PCRs were carried out in a total volume of 10 μl containing 2 μl of diluted cDNA, 200 nM *PPO1, 2* (2 primer pairs, one before and one after the CuA-binding domain)*, 3, 5*, and *6* gene primers (Table S2) and 5 μl of 2x Fast SYBR Green PCR Master Mix (Applied Biosystems, CA, USA) in an optical 96-well plate using a StepOnePlusTM Real-Time PCR System (Applied Biosystems, CA, USA). The qRT‒PCR temperature cycle was set at 95 °C for 10 min, followed by 40 cycles of 95 °C for 10 s, 58 °C for 15 s, and 72 °C for 20 s. The transcript levels of the PPO genes were compared by the 2^-ΔΔCt^ method. The *S. melongena* 18S gene was used as the endogenous reference for normalization and calculation of the fold change. All qPCR primers (Table S2) were designed by [https://www.idtdna.com/pages/tools/primerquest? returnurl=%2FPrimerquest%2FHome %2FIndex](https://www.idtdna.com/pages/tools/primerquest?%20returnurl=%2FPrimerquest%2FHome%20%2FIndex). Three technical replicates were analyzed for three biological replicates of each sample.

**Statistical analysis**

All analyses were repeated three times independently. The measurement of the phenotypic features was the mean of 5 replicates. The relative expression of the genes and standard error values were analyzed using the Microsoft Excel program. All numerical values are presented as the means±SEs. Statistical differences in the measurements between edited and nonedited plants were calculated using Student’s t-test.

**References**

Arnnok P, Ruangviriyachai C, Mahachai R, Techawongstien S, Chanthai S (2010) Optimization and determination of polyphenol oxidase and peroxidase activities in hot pepper (*Capsicum annum* L.) pericarp. Int Food Res J 17:385-392

Dutta I, Kottackal M, Tumimbang E, Tajima H, Zaid A, Blumwald E (2013) Sonication-assisted efficient *Agrobacterium*-mediated genetic transformation of the multipurpose woody desert shrub *Leptadenia pyrotechnica*. Plant Cell Tiss Organ Cult 112:289–301

Murashige T, Skoog F (1962) A revised medium for rapid growth and bioassays with tobacco tissue cultures. Physiol Plant 5:473–497
