# Supplementary material for: Hidden pleiotropy of agronomic traits uncovered by CRISPR-Cas9 mutagenesis of the tyrosinase CuA-binding domain of the polyphenol oxidase 2 of eggplant
Source: Plant Cell Rep. 2023 Feb 2;42(4):825–8. doi: 10.1007/s00299-023-02987-x (PMC10119049; doi:10.1007/s00299-023-02987-x)
Supplement: Supplementary file 2 — Supplementary file2 (PPTX 3014 KB) [file 299_2023_2987_MOESM2_ESM.pptx]

## Slide 1
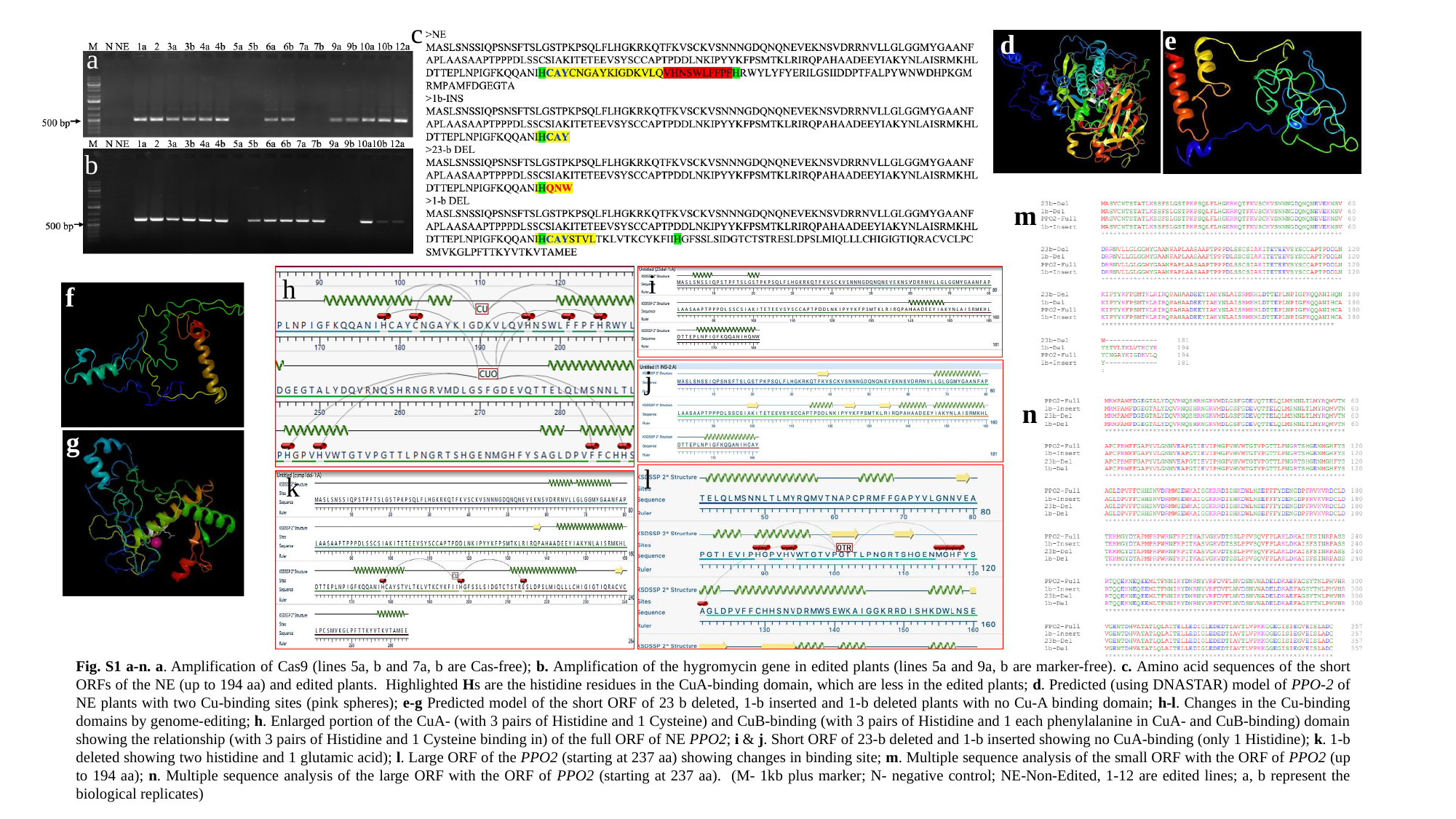

c
e
d
a
b
m
n
i
h
j
l
k
f
g
Fig. S1 a-n. a. Amplification of Cas9 (lines 5a, b and 7a, b are Cas-free); b. Amplification of the hygromycin gene in edited plants (lines 5a and 9a, b are marker-free). c. Amino acid sequences of the short ORFs of the NE (up to 194 aa) and edited plants. Highlighted Hs are the histidine residues in the CuA-binding domain, which are less in the edited plants; d. Predicted (using DNASTAR) model of PPO-2 of NE plants with two Cu-binding sites (pink spheres); e-g Predicted model of the short ORF of 23 b deleted, 1-b inserted and 1-b deleted plants with no Cu-A binding domain; h-l. Changes in the Cu-binding domains by genome-editing; h. Enlarged portion of the CuA- (with 3 pairs of Histidine and 1 Cysteine) and CuB-binding (with 3 pairs of Histidine and 1 each phenylalanine in CuA- and CuB-binding) domain showing the relationship (with 3 pairs of Histidine and 1 Cysteine binding in) of the full ORF of NE PPO2; i & j. Short ORF of 23-b deleted and 1-b inserted showing no CuA-binding (only 1 Histidine); k. 1-b deleted showing two histidine and 1 glutamic acid); l. Large ORF of the PPO2 (starting at 237 aa) showing changes in binding site; m. Multiple sequence analysis of the small ORF with the ORF of PPO2 (up to 194 aa); n. Multiple sequence analysis of the large ORF with the ORF of PPO2 (starting at 237 aa). (M- 1kb plus marker; N- negative control; NE-Non-Edited, 1-12 are edited lines; a, b represent the biological replicates)

## Slide 2
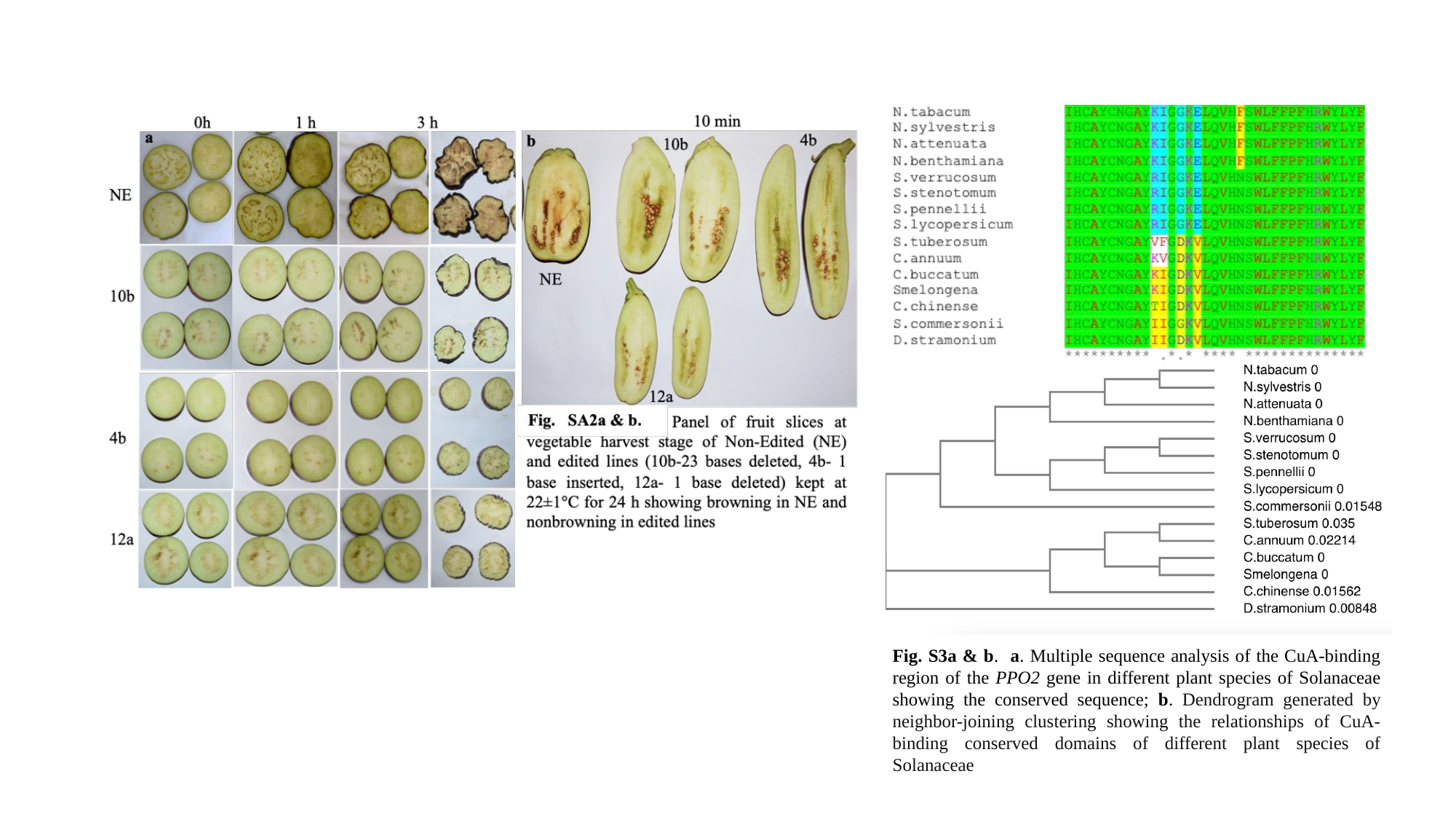

Fig. S3a & b. a. Multiple sequence analysis of the CuA-binding region of the PPO2 gene in different plant species of Solanaceae showing the conserved sequence; b. Dendrogram generated by neighbor-joining clustering showing the relationships of CuA-binding conserved domains of different plant species of Solanaceae

## Slide 3
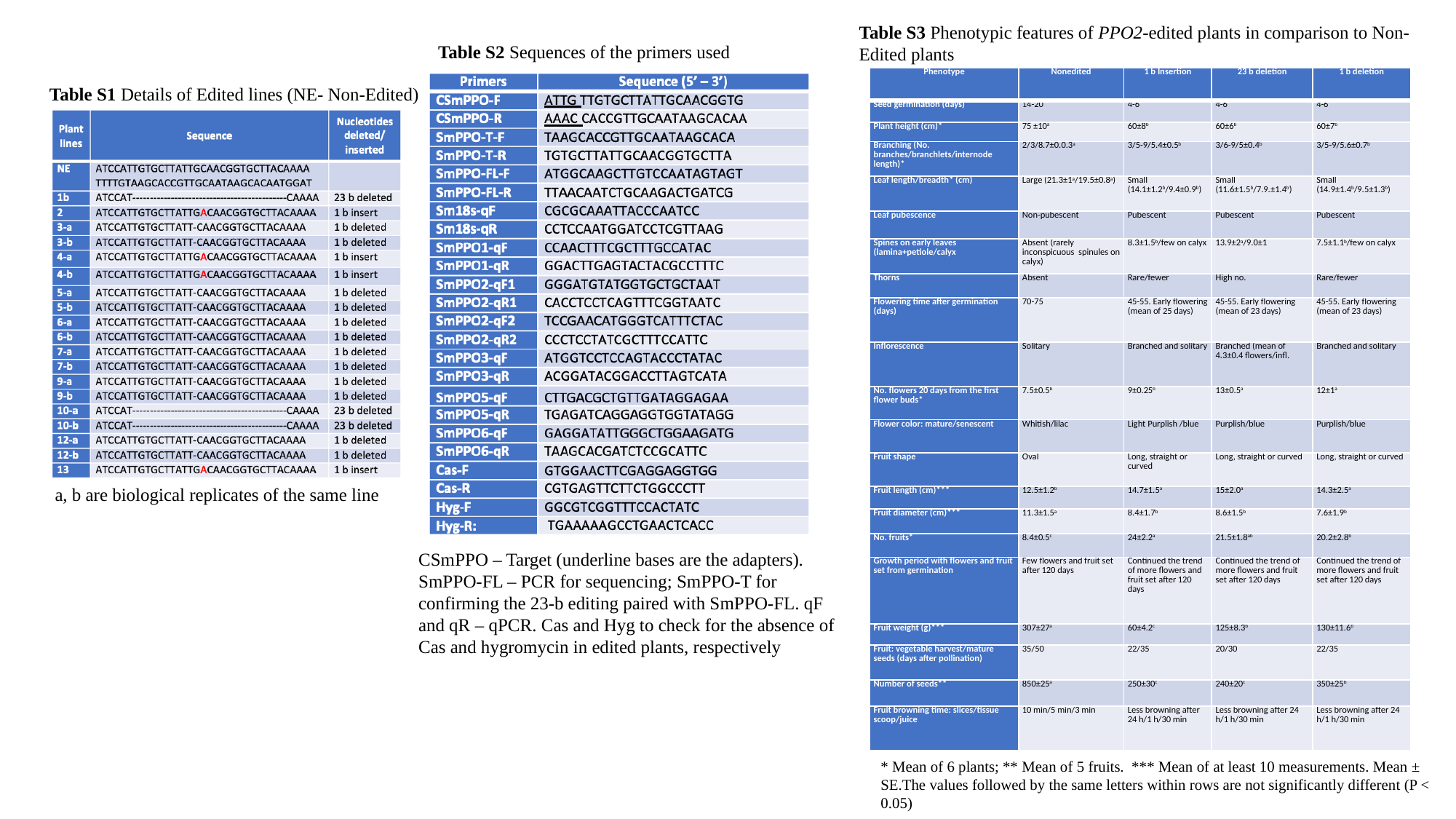

Table S3 Phenotypic features of PPO2-edited plants in comparison to Non-Edited plants
Table S2 Sequences of the primers used
| Phenotype | Nonedited | 1 b Insertion | 23 b deletion | 1 b deletion |
| --- | --- | --- | --- | --- |
| Seed germination (days) | 14-20 | 4-6 | 4-6 | 4-6 |
| Plant height (cm)\* | 75 ±10a | 60±8b | 60±6b | 60±7b |
| Branching (No. branches/branchlets/internode length)\* | 2/3/8.7±0.0.3a | 3/5-9/5.4±0.5b | 3/6-9/5±0.4b | 3/5-9/5.6±0.7b |
| Leaf length/breadth\* (cm) | Large (21.3±1a/19.5±0.8a) | Small (14.1±1.2b/9.4±0.9b) | Small (11.6±1.5b/7.9.±1.4b) | Small (14.9±1.4b/9.5±1.3b) |
| Leaf pubescence | Non-pubescent | Pubescent | Pubescent | Pubescent |
| Spines on early leaves (lamina+petiole/calyx | Absent (rarely inconspicuous spinules on calyx) | 8.3±1.5b/few on calyx | 13.9±2a/9.0±1 | 7.5±1.1b/few on calyx |
| Thorns | Absent | Rare/fewer | High no. | Rare/fewer |
| Flowering time after germination (days) | 70-75 | 45-55. Early flowering (mean of 25 days) | 45-55. Early flowering (mean of 23 days) | 45-55. Early flowering (mean of 23 days) |
| Inflorescence | Solitary | Branched and solitary | Branched (mean of 4.3±0.4 flowers/infl. | Branched and solitary |
| No. flowers 20 days from the first flower buds\* | 7.5±0.5b | 9±0.25b | 13±0.5a | 12±1a |
| Flower color: mature/senescent | Whitish/lilac | Light Purplish /blue | Purplish/blue | Purplish/blue |
| Fruit shape | Oval | Long, straight or curved | Long, straight or curved | Long, straight or curved |
| Fruit length (cm)\*\*\* | 12.5±1.2b | 14.7±1.5a | 15±2.0a | 14.3±2.5a |
| Fruit diameter (cm)\*\*\* | 11.3±1.5a | 8.4±1.7b | 8.6±1.5b | 7.6±1.9b |
| No. fruits\* | 8.4±0.5c | 24±2.2a | 21.5±1.8ab | 20.2±2.8b |
| Growth period with flowers and fruit set from germination | Few flowers and fruit set after 120 days | Continued the trend of more flowers and fruit set after 120 days | Continued the trend of more flowers and fruit set after 120 days | Continued the trend of more flowers and fruit set after 120 days |
| Fruit weight (g)\*\*\* | 307±27a | 60±4.2c | 125±8.3b | 130±11.6b |
| Fruit: vegetable harvest/mature seeds (days after pollination) | 35/50 | 22/35 | 20/30 | 22/35 |
| Number of seeds\*\* | 850±25a | 250±30c | 240±20c | 350±25b |
| Fruit browning time: slices/tissue scoop/juice | 10 min/5 min/3 min | Less browning after 24 h/1 h/30 min | Less browning after 24 h/1 h/30 min | Less browning after 24 h/1 h/30 min |
Table S1 Details of Edited lines (NE- Non-Edited)
a, b are biological replicates of the same line
CSmPPO – Target (underline bases are the adapters). SmPPO-FL – PCR for sequencing; SmPPO-T for confirming the 23-b editing paired with SmPPO-FL. qF and qR – qPCR. Cas and Hyg to check for the absence of Cas and hygromycin in edited plants, respectively
* Mean of 6 plants; ** Mean of 5 fruits. *** Mean of at least 10 measurements. Mean ± SE.The values followed by the same letters within rows are not significantly different (P < 0.05)
.
